# Immature Cryopreserved Ovary Restores Puberty and Fertility in Mice without Alteration of Epigenetic Marks

**DOI:** 10.1371/journal.pone.0001972

**Published:** 2008-04-16

**Authors:** Frédérique Sauvat, Carmen Capito, Sabine Sarnacki, Catherine Poirot, Anne Bachelot, Geri Meduri, Luisa Dandolo, Nadine Binart

**Affiliations:** 1 Inserm, Unit 845, Paris, France; 2 Univ Paris-Descartes, Faculty of Medicine René Descartes, Paris 5-Necker site, UMR-S845, Paris, France; 3 AP HP, Department of Pediatric Surgery, Hospital Necker Enfants-Malades, Paris, France; 4 AP HP, Unit of Biology of Reproduction, GH Pitié-Salpétrière, Paris, France; 5 AP HP, Department of Endocrinology and Reproductive Medicine, GH Pitié-Salpétrière, Paris, France; 6 Inserm, Unit 693, Le Kremlin Bicêtre, France; 7 AP HP, Department of Molecular Biochemistry, GH Bicêtre, Le Kremlin Bicêtre, France; 8 Department of Genetics and Development, Institut Cochin, University Paris Descartes, CNRS UMR 8104, Inserm U 567, Paris, France; University of Kansas Medical Center, United States of America

## Abstract

**Background:**

Progress in oncology could improve survival rate in children, but would probably lead to impaired fertility and puberty. In pre-pubertal girls, the only therapeutic option is the cryopreservation of one ovary. Three births have been reported after reimplantation of cryopreserved mature ovary. Conversely, reimplantation of ovary preserved before puberty (defined as immature ovary) has never been performed in humans.

**Methodology/Principal Findings:**

In order to analyze ovarian function, we performed transplantation using fresh or cryopreserved immature grafts in pre-pubertal or adult mice. Puberty as well as cyclic hormonal activity was restored. All follicle populations were present although a significant reduction in follicle density was observed with or without cryopreservation. Although fertility was restored, the graft is of limited life span. Because ex vivo ovary manipulation and cryopreservation procedure, the status of genomic imprinting was investigated. Methylation status of the H19 and Lit1 Imprinting Control Regions in kidney, muscle and tongue of offsprings from grafted mice does not show significant alteration when compared to those of unoperated mice.

**Conclusions/Significance:**

These results demonstrate that immature ovarian grafting can restore spontaneous puberty and fertility. However, these data suggest that follicle depletion leads to premature ovarian failure. This study addresses the very important epigenetics issue, and provides valuable information to the study of ovarian transplantation suggesting that these procedures do not perturb normal epigenetics marks. These results are highly relevant to the reimplantation question of immature cortex in women.

## Introduction

Malignant childhood cancers represent 1% of all cancers, which means, for example, a pediatric cancer incidence in USA of 122 per million children [1] or in France 1800 new pediatric patients each year (700 between 15 to 19 years old) [2]. In 1973, the five-year survival rate after pediatric cancer was 50.4% [3]. Since 1990, progress in pharmacology has allowed a five-year survival rate of nearly 75% [4]. Therefore in 2010, one young adult (15–45 years old) in 250 to 715 will be a pediatric cancer survivor [5], [6]. However these therapeutical advances are associated with long-term morbidities, especially regarding ovarian failure [7], responsible for puberty delay [8] and infertility [9].

In adults, embryo freezing remains the best choice to preserve fertility of women with a partner [10]. The other options are oocyte cryopreservation or ovarian cortex cryopreservation [11], before treatment [12]. In pre-pubertal girls, the alternative is the cryopreservation of one ovary, a protocol currently proposed in some countries to children before the sterilizing treatment [13]. Although births have been reported after reimplantation of either cryopreserved [14]–[18] or fresh adult ovarian cortex [19]–[21], the efficiency of pre-pubertal ovarian cortex for the restoration of fertility and endocrine function is still unknown. Particularly, the timing of reimplantation must deal with the major aim of this strategy, i.e. restoration of fertility. However, induction of puberty by early grafting should also be questioned. In animal models (ewes, mice, rats, rabbits), the use of adult cryopreserved ovaries has been largely reported and only two experiments describe the reimplantation of fetal or neonatal ovarian cortex [22], [23]. Another point of interest is the epigenetic changes in animals conceived by assisted reproductive technologies (ART). In particular, genomic imprinting refers to an epigenetic marking of certain genes, resulting in monoallelic expression in a parent of origin-dependent manner. Imprinted genes are characterized by Differentially Methylated Regions (DMR). The allele specific DNA methylation is established during gametogenesis (after puberty in oocytes) and must be maintained during pre-implantation development when the rest of the genome is subjected to a wave of demethylation [24]. At the distal end of human chromosome 11p15.5 and mouse chromosome 7, two clusters of imprinted genes are under the control of imprinting control regions (ICR). The *H19-Igf2* region is controlled by the *H19* ICR, methylated on the paternal allele [25], [26]. The second adjacent cluster contains several maternally expressed genes that include *Cdknc1* and *Kcnq1*. This region harbors a single paternally expressed gene *Lit1* (or *Kcnq1ot1*) with a CpG island called KvDMR1 which is methylated on the maternal chromosome during oogenesis [27]–[29]. Aberrant genomic imprinting induces numerous genetic disorders. As an example, patients with Beckwith-Wiedemann Syndrome (BWS), linked to chromosome 11p15.5, have abnormal hypermethylation of the maternal *H19* ICR, leading to higher expression of *IGF2* with an overgrowth syndrome and increased cancer risk [30]–[34]. A slight increase in imprinting anomalies has also been reported in children conceived by assisted reproductive technology with a higher incidence of BWS, Prader-Willi or Angelman syndrome [35]–[40]. The epimutation reported in BWS after ART involves a loss of methylation at the maternally methylated KvDMR1 [36]. Because the *H19-Igf2* and *Lit1* regions appear to be sensitive to external modifications [41], [42], *H19* ICR and *Lit1* KvDMR1 methylation were used in this study to evaluate epigenetic changes in pups born after ovarian cryopreservation.

In this report, we performed orthotopic ovarian transplantation using fresh and/or cryopreserved whole ovaries from non-pubescent mice and demonstrated the efficiency of these immature (defined as preserved before puberty) cryopreserved ovaries to restore both endocrine function and fertility even in immature recipients. We also focused on the methylation status of the *H19* ICR and *Lit1* KvDMR1 from grafted mice and were able to show absence of significant difference compared with non manipulated mice, suggesting that cryopreservation and/or ovarian transplantation do not interfere with proper establishment of genomic imprinting.

## Methods

### Animals

Mice of 129 Sv/J genetic background were housed under normal laboratory conditions in a 12-h light, 12-h dark cycle (0700–1900), in the animal facility of the Necker Faculty. The temperature was controlled (21°C), and the animals had free access to tap water and standard pelleted animal food. Immature ovaries were obtained from mice operated before three weeks of age at day 18 after birth (the regular age at puberty is of 5–6 weeks). Recipients between three and four weeks were considered as immature and after 2 months of age as adult mice. All procedures were approved by the Necker Faculty Lab-Animal Care and Use Committee. Controls groups contained non manipulated animals and animals with unilateral ovariectomy.

### Orthotopic ovarian transplantation

According to the groups defined in Table 1, 86 mice were operated. Anesthesiology was performed with solution containing 150 µl of Xylazine (Rompun®, Bayer Health Care), 600 µl of Ketamine (Imalgene®100, Merial) and physiological injectable serum (3 ml). Dose of 0.1 ml for 10 g of mice weight was injected intra-peritoneally. Ovarian grafts were obtained by removal of one entire ovary and placed in Brahma® 1 solution (CryoBioSystem, l'Aigle, France) to use freshly (n = 51) or after cryopreservation (n = 35). Ovarian transplantation was performed by bilateral posterior approach after removal of both ovaries from the bursa surrounding ovaries and replacement of one entire ovary (fresh or cryopreserved) near the oviduct, in the ovarian bursa [43]. Skin was sutured after the operation. Surgery was performed under Zeiss surgical microscope (Carl Zeiss, Oberikochen, Germany) at room temperature. Intact ovary was either frozen within 30 min of dissection or grafted into the ovarian bursa within 20 min of dissection.

**Table 1:** Groups of transplanted mice and cyclic hormonal activity.

	Graft's status	Donor's status	Recipient's status	n	Histology	Fertility	Recovery of cycle	Length of cycle
**NNF**	Fresh (F)	Non mature (N)	Non mature (N)	27	5	22	16.6±2.5	5.1±0.9
**NAF**		Non mature (N)	Adult (A)	16	4	12	17.5±3.3	4.5±1.0
**AAF**		Adult (A)	Adult (A)	15	7	8	15.6±2.0	5.2±1.1
**NNC**	Cryopreserved (C)	Non mature (N)	Non mature (N)	11	3	8	10.2±0.4	5.4±0.8
**NAC**		Non mature (N)	Adult (A)	9	6	3	11.5±0.5	6.5±0.7
**AAC**		Adult (A)	Adult (A)	8	4	4	12.6±1.5	4.6±0.8
**Total**				**86**	**29**	**57**		

Non-pubertal mouse, 18 day-old was defined as immature and grafted in non-mature (N) or adult mice (A), using cryopreserved (C) or fresh (F) graft. Grafted mice were divided in two groups: mice sacrificed one month after grafting for histological study and the others mated with stud male, to study fertility. n, number of animals/group used for analysis.

Recovery of cycle corresponded to the delay between transplantation and the vaginal opening in non mature recipients and to the delay between transplantation and the recovery of cyclic activity in adult recipients. Length of cycle was defined as the delay in days between two estrus. Data are expressed in days, as mean±SEM.

### Ovarian cryopreservation

Ovaries were cryopreserved using the same protocol as described by Gosden *et al*. [44]. Whole ovary tissue was equilibrated at 4°C for 30 min in cryogenic vials containing Brahma® 1 solution with 10% bovine calf serum and 1.5 M dimethylsulfoxide (DMSO). The vials were transferred to a programmable freezer (Planner Products, Cryobiosystem) and cooled at 2°C/min for seeding and followed by 0.3°C/min to −40°C and subsequently at 10°C/min to −140°C. Vials were stored in liquid nitrogen, for approximately 2 to 4 weeks. Before grafting, tissues were rapidly warmed in air at room temperature for 30 s and then immersed in water at 30–35°C for 5 minutes. The ovaries were removed from the cryovials and placed successively in 4 solutions containing decreasing DMSO concentrations. After washing, ovaries were placed in Leibovitz-L15 medium, at room temperature, and grafted within 15 min of thawing.

### Evaluation of onset of puberty and estrous tracking

Female mice were observed daily, after ten days of grafting, for appearance of vaginal opening. Once this occurred, daily histological analysis of vaginal smears was taken. The vaginal wall was washed using 20 µl of PBS and cells smeared onto a clean glass slide. The stage of estrous cycle was determined from the cell types observed in the vaginal smear [45]. Estrous cycle was determined as the interval between onset of one estrous and the next estrous event.

### Breeding test

After recovery of cycle activity, 57 grafted mice were paired with mature males of the same genetic background. Control group comprised unilateral ovariectomized mice and WT mice. Vaginal plugs were inspected daily. The date of birth and size of each litter was recorded. When a female failed to produce a litter within 90 days of the initial mating, the male was replaced. If no litter or visible signs of pregnancy (by daily weight increase) were observed after three months of mating or last pregnancy, the female was autopsied and examined for ovarian tissue.

A secondary NNF group of 10 mice was sacrificed 5.5 days after the observation of the first plug. Implantation sites (IS) were visualized by an i.v. injection (0.1 ml/mouse) of Chicago blue B dye solution (1% in saline), and the number of ISs demarcated by distinct blue bands was recorded.after injection of 0.1 ml of Chicago Blue. Number of IS in uteri and corpora lutea by ovary was counted then compared.

### Histology and immunohistology

Early ovarian histology was obtained by sacrifice of 29 mice from different groups 30 days after grafting. Removed ovaries were placed in formalin then embedded in paraffin wax, serially sectioned at 5 µm and stained with haematoxylin and eosin. The sections were examined for the presence of follicles and corpora lutea. Sections were also stained with periodic acid Schiff (PAS) for apoptosis study. In same mice, proliferation was tested by immunohistochemical staining of PCNA and vascularisation by α-actin smooth muscle staining on 5 µm thick sections from formalin-fixed, paraffin-embedded tissue blocks. Briefly, the sections were de-paraffinized and boiled in microwave for 2×5 min in 0.01 M citrate buffer pH 6, then cooled to room temperature for 30 min. For PCNA immunostaining, polyclonal rat anti-mouse antibody (Santa Cruz biotechnology) was used at a 1∶250 dilution. For α-actin smooth muscle staining, polyclonal anti-mouse antibody (Sigma) was used at a 1∶300 dilution. Immunostaining with anti-3β-HSD was done at a dilution of 1∶3000 [46]. DAKO TechMate™ 500 Plus staining system was used following the manufacturer's instructions. Negative controls were processed in parallel by replacing the primary antibody by buffer.

### Follicular density

In slides obtained from 29 mice sacrificed early, follicular density was calculated by reporting the total number of follicles by the surface unit [47]. Ovaries fixed in 4% buffered formaldehyde and included in paraffin were serially sectioned at a thickness of 5 µm, then sections were stained and were examined serially for the presence of follicles and corpora lutea by ovary. To avoid counting follicles more than once, follicles were counted in the section where the dark-staining nucleolus was seen within the nucleus of the oocyte using the Lucia software. Results were pooled by group type and compared to unmanipulated ovaries. Results are expressed as number by surface unit.

### Uterus macroscopical analysis and mammary gland whole mount

Uteri and mammary glands from manipulated mice having received immature cortex in pre-pubertal or adult recipients were analyzed one month after grafting or during pregnancy (mammary gland), and compared with non-pubertal unmanipulated mice. Inguinal mammary glands for whole-mount analysis were spread on a glass slide and fixed overnight in 25% acetic acid/75% ethanol. The samples were washed in 70% ethanol and stained overnight in carmine (Sigma-Aldrich), dehydrated in ethanol and xylene, and mounted with coverslip.

### Methylation status of H19 ICR and Lit1 KvDMR1 in pups issued from grafted mice

Epigenetic status was studied in pups from grafted mice, included in the six groups previously described and in mice issued from non manipulated mice, by collecting kidney, muscle and tongue at 21 days of age. The methylation status of these *H19* ICR was followed at early stages after birth by analyzing nine unmanipulated mice sacrificed at 5, 10 and 15 days of life.

Genomic DNA was prepared from tissues by standard proteinase K digestion and phenol–chloroform extraction methods. For *H19* ICR, genomic DNA from each sample was digested with *Sac1* and the methylation-sensitive enzyme *Hha1*, separated on 1% agarose gel, transferred to Hybond N+ (GE Healthcare) in 0.4 M NaOH and hybridized to the CTCF3 specific probe. The probe was obtained by PCR with sense primer: 5′ctgttatgtgcaacaagggaa and anti-sense primer: 5′ggtcttaccagccactga. The upper band (3.8 kb) is methylated and corresponds to the paternal allele. The lower band (0.3 kb) is unmethylated and corresponds to the maternal allele. Methylation indexes were determined by densitometry of autoradiographs using a Storm PhosphoImager (Molecular Dynamics, Sunnyvale, USA). Results were expressed as the ratio (MI) between fragment intensity of the upper methylated band compared to the sum of fragment intensity of upper band and lower unmethylated band. In physiological status, MI is of 0.5.

For *Lit1* KvDMR1, genomic DNA was digested with *Apa1* and the methylation-sensitive enzyme *Sma1* and probed with a *KvDMR1* fragment obtained by PCR using sense primer: 5′cactggagctgaaaccgaatcg and anti-sense primer: 5′aaaacaggtgcagaaatgg. A maternal methylated 2.6 kb fragment and a paternal 0.9 kb unmethylated fragment were detected for this DMR.

### Statistical analysis

Statview software was used for statistical analysis. ANOVA (multiple comparisons) was used. The significance was set at p<0.05, and the values were presented as mean±SEM as specified.

## Results

### Onset of puberty and restoration of hormonal activity

A total of 86 female mice successfully underwent orthotopic ovarian transplantation. In 63 animals ovarian grafts originated from mice of less than three weeks of age considered as immature donor mice (pre-pubertal). Fresh (F) (n = 43) or cryopreserved (C) (n = 20) immature ovarian grafts were transplanted either in non mature (N) (n = 38) or in adult (A) (n = 25) mice. The control group consisted of 23 adult mice receiving fresh (n = 15) or cryopreserved (n = 8) adult ovarian grafts (Table 1). After surgery, the occurrence of vaginal opening and onset of estrus cycle were observed as an external indicator of functional graft in all groups (Table 1). When the recipient was pre-pubertal at time of grafting, occurrence of cycle was considered as the sign of pubertal instauration. The study of estrus cycle was tracked by analysis of vaginal cytology for a period of 21 days. The mean age of puberty onset occurred between 5 and 6 weeks of age in immature grafted mice as well as in unoperated control mice. Recovery of hormonal activity occurred after 14.9±2.8 days, without any statistical difference between groups. In particular, no difference was observed between the groups of transplanting immature ovaries to immature animals (NNF and NNC) and immature ovaries to adult animals (NAF and NAC) as well as in immature ovaries to adult animals (NAF and NAC) compared to adult ovaries to adult animals (AAF and AAC). Data indicated that length of estrous cycle was similar whatever the age of donor and recipient and the status of graft (fresh or cryopreserved) corresponding to a number of 4–5 estrous cycles during this experimental period.

### Ovarian histology

**Figure 1:**
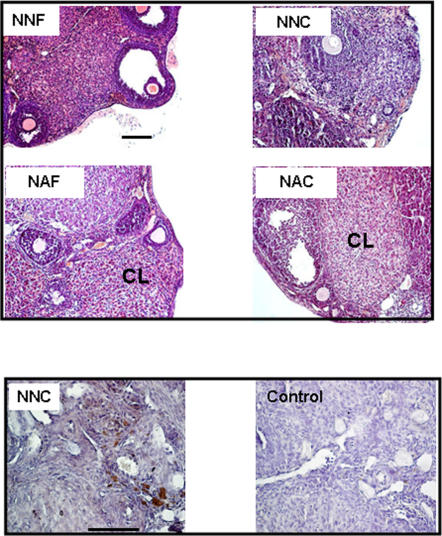
Histological analysis of ovary after one month of grafting and vascularization analysis. Immature ovarian cortex was grafted in non-pubertal mice freshly (NNF) either after cryopreservation (NNC) or in adult mice freshly (NAF) or after cryopreservation (NAC). Corpus luteum (CL) signs ovulation process. All images are at the same magnification. Representative slide of one ovary from NNC group is shown after immunohistochemical detection of vascularization using α-actin smooth muscle antibody as compared with control (without primary antibody). Scale bar, 100 µm

**Figure 2:**
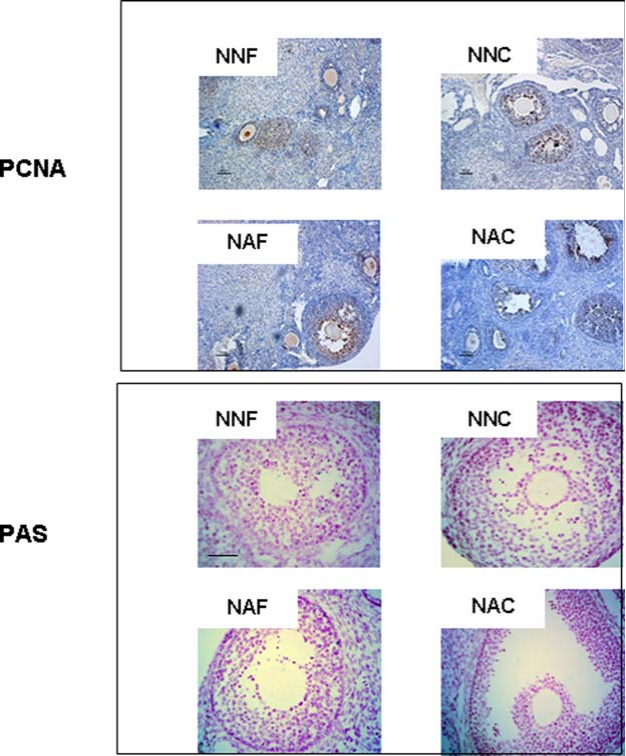
Follicular proliferation and apoptosis. Cell proliferation was studied using PCNA antibody and apoptosis using PAS staining one month after grafting. Immature ovarian cortex was grafted in non-mature mice freshly (NNF) or after cryopreservation (NNC) or in adult mice freshly (NNF) or after cryopreservation (NNC). All images are at the same magnification. Scale bar, 50 µm

Examination of ovaries after one month of grafting showed the presence of follicles at all developmental stages and corpora lutea (CL) in all groups (Figure 1). The presence of CL indicated that ovulation had occurred. Macroscopic analysis of the whole graft showed the re-establishment of a network of blood vessels demonstrating that revascularization of the graft took place in all analyzed groups, as assessed by the presence of capillaries. Immunostaining using α-actin smooth muscle antibody, a marker for blood perivascular smooth muscle cells, also attested the presence of vascularisation in layers surrounding the granulosa cells. In larger follicles, this layer became hyperemic and numerous small blood vessels could be seen as well as some stromal vessels in the medullary region of transplanted ovary (Figure 1). Analysis of the proliferation rate of follicular cells by PCNA immunostaining in follicles demonstrated active proliferation in all experimental groups. Proliferation was not modified by the age of donors, neither by the process of cryopreservation. Moreover, apoptosis of follicles analyzed by PAS staining demonstrated a similar pattern of cellular death in all groups (Figure 2).

### Follicular density

**Figure 3.Follicle:**
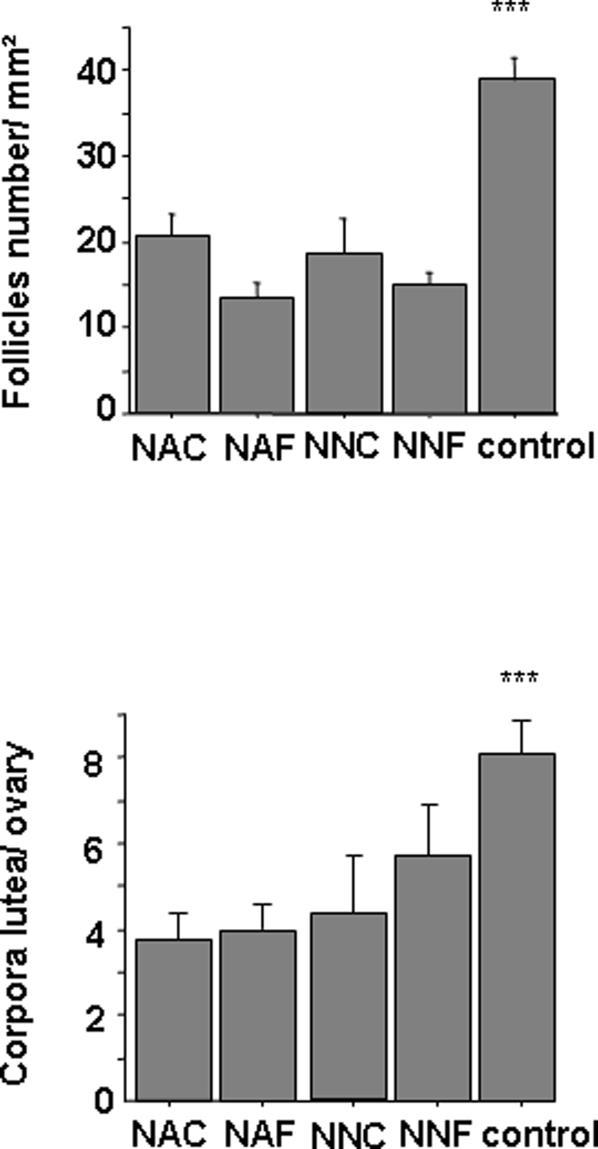
density and number of corpora lutea by ovary in each group one month after grafting. n = 5 mice/group. Data are expressed as mean density±SEM. The asterisks indicate significant differences between groups of manipulated mice and unmanipulated mice (control) (p<0.001)

After transplantation into unmanipulated recipient mice, the ovaries developed many preantral, antral follicles and corpora lutea. The evaluation of follicular density showed no difference between groups of mice receiving immature grafts but was significantly decreased as compared to unmanipulated control mice (16.14 versus 38 follicles/mm^2^) (Figure 3). The follicular loss was thus clearly linked to grafting related ischemia rather than cryopreservation process or recipient age. The mean number of corpora lutea by group was also significantly decreased as compared with unmanipulated mice.

### Breeding tests and size of litters

**Figure 4:**
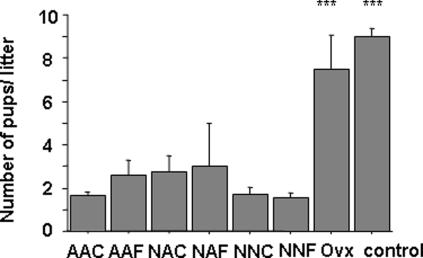
Number of pups by litter in each group. Size of the litter was recorded in each group as compared to unmanipulated mice (control) and to mice after unilateral ovariectomy (Ovx). The asterisks indicate significant differences (p<0.001) between manipulated groups and control groups. All data are expressed as mean±SEM.

Daily observations of mice revealed that 100% of the transplanted (n = 57) and unmanipulated control mice (n = 15) mated, as indicated by the appearance of vaginal plugs, a sign of normal sexual behavior. After a normal length of pregnancy, 25 to 50% of grafted mice gave birth to offspring (Table 2). Mean number of gestations by mouse during the observation period was between 1.5 and 2.5 in all mice receiving immature ovary. The last gestation occurred between 3 and 8.75 months after grafting. This is very early in lifespan and statistically significant (p<0.005) when compared to the mean length of reproductive life in unmanipulated control mice. These results indicate that the transplantation process, even with immature ovaries, induces premature ovarian failure. In all manipulated groups, the mean litter size was significantly decreased compared to unmanipulated control mice (2.0±0.5 versus 9.1±0.6 pups) (Figure 4). Since reimplantation was done with only one ovary, a new control group of mice undergoing unilateral ovariectomy was generated and this group gave rise to a litter size similar to unmanipulated control mice. Comparison of grafted groups with both control groups (unmanipulated and unilaterally ovariectomized) was thus significantly different (Figure 4).

**Table 2:** Litter sizes following the first, second and third pregnancies, in each group and secondary infertility.

		1st pregnancy		2nd pregnancy		3rd pregnancy		Secondary Infertility
	Vaginal plug	Litter size	%/all	Litter size	%/all	Litter size	%/all	
**NNF**	22/22	1.7±0.2	36%	1.6±0.3	13.3%	1.0	4.5%	5.5±2.1
**NNC**	12/12	1.0±0	33%	1.6±0.3	25%	2.0±1	16.6%	5.7±1.3
**NAF**	8/8	4.0±3.0	25%	1.0	12.5%	-	-	3.0±1.7
**NAC**	8/8	1.5±0.5	50%	5.0±0.5	37.5%	1.0	12.5%	8.7±2.6
**AAC**	4/4	1.5±0.5	50%	2.0	25%	2.0	25%	5.5±2.0
**AAF**	3/3	5.0	33%	1.0	33%	1.0	33%	8.0
**WT**	15/15	9.1±0.6	100%	9.4±0.8	100%	9.0±1	100%	>18

Vaginal plug, number of mice after mating/number of studied mice, by group. %/all, pregnancy rate of females mated. Litter size: mean of pups by female. Secondary infertility was defined as the date, in months after grafting of the ultimate gestation. All data are expressed as mean±SEM.

In NNF group of mice sacrificed 5.5 days after the appearance of the plug, a significant difference existed between the number of implantation sites (0.8±0.32 by mouse) and the number of corpora lutea seen in the ovary (5.5±0.74 by ovary) suggesting a failure of early implantation.

Out of 57 grafted females exhibiting a plug, only 21 gave birth to pups. In order to understand the absence of pregnancy in the 36 remaining mice, we performed an autopsy after three months of infertility. In 44% of infertile mice (16/36), ovaries were macroscopically and histologically normal. In other cases, we found a regression of transplants in 36% of cases (13/36) as well as misallocation of the transplanted tissue outside bursa ovary in 19% of cases (7/36). The rate of fertility was thus recalculated by excluding mice with an anatomical cause of failure (20/36) and was found to be between 40 and 80%.

### Uterine and mammary gland whole mount assessment

**Figure 5:**
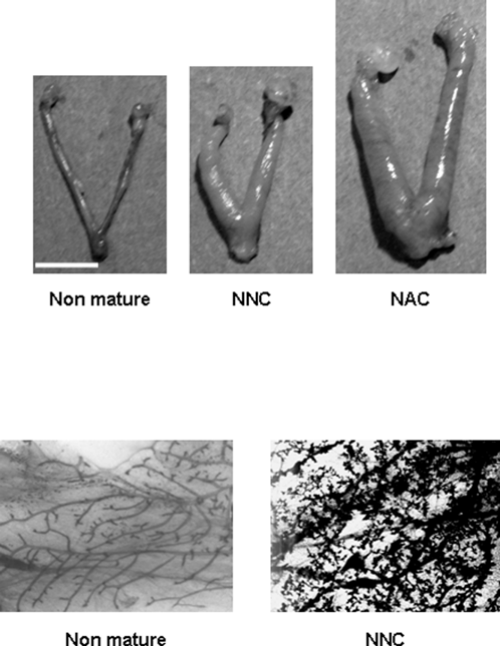
Representative macroscopic images of the uteri and mammary gland. Uterus of non-mature unmanipulated mouse is compared to those of non-pubertal mouse grafted with immature cortex (NNC), two months after transplantation and of pubertal mice grafted with immature cortex (NAC), six months after transplantation. All images are at the same magnification. Scale bar, 1 cm. Representative whole mount of mammary glands from non mature mouse is compared to the mammary gland from a pre-pubertal mouse grafted with immature cortex (NNC) at mid pregnancy.

The macroscopical aspect of the uterus in NNF mice after onset of puberty was similar to the one observed in adult unmanipulated mice and significantly different from pre-pubertal unmanipulated control mice (Figure 5). This demonstrated the efficiency of grafted ovaries to synthesize steroids for uterus impregnation. Whole-mount analysis of mammary glands in grafted mice showed normal ducts and small terminal end buds, similar to those observed in pubertal unmanipulated control mice as well as normal development of the mammary gland during the second half of gestation (Figure 5).

### Analysis of long-term follicle depletion

**Figure 6:**
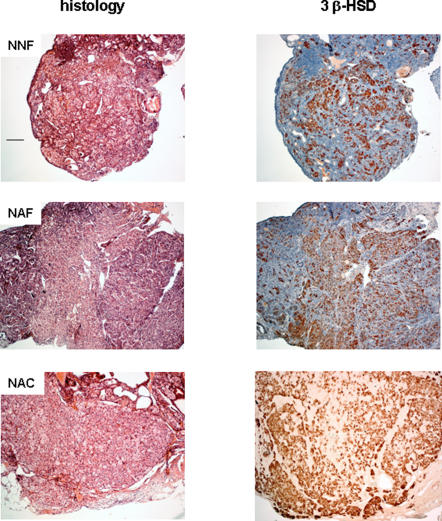
Representative histological and immunohistochemical analysis for 3 β-HSD of ovaries at one year after grafting. Mice were grafted with fresh (NNF, NAF) or cryopreserved (NAC) immature cortex ovaries. Recipients were non-mature (NNF) or adult mice (NAF and NAC). Scales bar 100 µm.

In transplanted mice, analysis of ovarian graft after the last gestation showed complete follicular depletion in all cases (Figure 6) with follicular impairment and numerous scarses resulting from follicular death and remnants of oocytes that have undergone atresia. Overall analysis showed the presence of interstitial cells with a steroidogenic appearance without any granulosa cells. Moreover, to evaluate the steroidogenesis ovarian sections were assessed by immunohistochemistry with P450scc, 3β-HSD and P450 C17 antibodies. In all groups 3β−HSD enzyme was detected in about 50% of the interstitial cells suggesting that steroidogenesis was still present (Figure 6).

### Methylation status of H19 ICR and Lit1 KvDMR1 in pups from grafted mice

**Figure 7:**
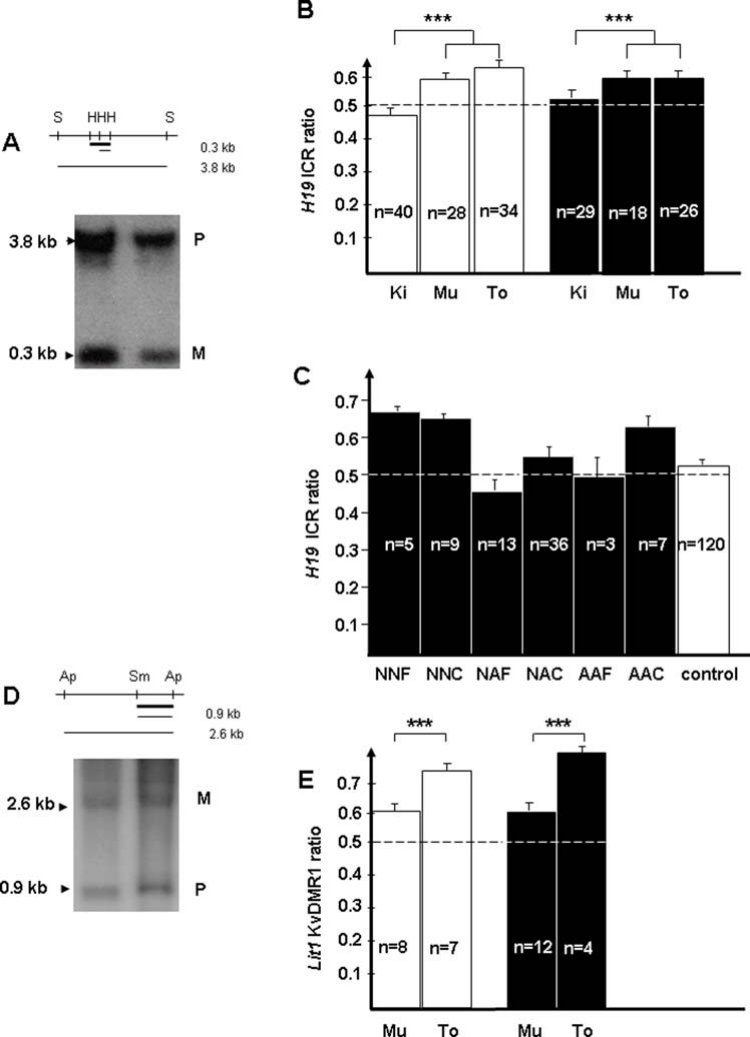
Methylation status of *H19* ICR and *Lit1* KvDMR1. (A) *H19* ICR methylation was determined by Southern blotting after digestion with *Sac I* (S) and *Hha1* (H) enzymes. The upper band (3.8 kb) corresponds to the paternal methylated allele (P) and the lower band (0.3 kb) corresponds to the maternal unmethylated allele (M). Probe is marked by thick black line. (B) *H19* ICR methylation was analyzed in DNA from three tissues: kidney (Ki), muscle (Mu), and tongue (To) in unmanipulated (white) or grafted animals (black). ***, p<0.001 for kidney compared to both other groups (muscle and tongue) (C) *H19* ICR ratio was represented in unmanipulated (control, white) or grafted animals (black) in three tissues according to the manipulated group. (D) *Lit1* KvDMR1 methylation was determined by Southern blotting after digestion with *Sma I* (Sm) and *Apa I* (Ap) enzymes. The upper band (2.6 kb) corresponds to the maternal methylated allele (M) and the lower band (0.9 kb) corresponds to the paternal unmethylated allele (P). Probe is marked by thick black line. (E) *Lit 1* KvDMR1 ratio was compared in muscle (Mu) and tongue (To) from unmanipulated (white bar) or grafted animals (black bar). ***, p<0.001 for tongue vs muscle.

Three different tissues were collected in 76 pups from grafted mice as well as in 52 unmanipulated pups representing the control group. Southern blotting of *SacI/HhaI* digested DNA followed by hybridization with an *H19* internal probe, allowed detection of an unmethylated maternal allele (0.3 kb) and a methylated paternal allele (3.8 kb) (Figure 7A). The methylation index (MI: methylated fragment/unmethylated + methylated fragment) was analyzed at 5, 10, 15 and 21 days after birth in kidney, muscle and tongue from control mice and no significant difference was found in control mice between these different time points (data not shown). The global methylation status of the three tissues was then evaluated in manipulated samples taken at 21 days and compared to control samples. A slight difference was observed between muscle and tongue DNA methylation (MI>0.6) compared to kidney DNA (MI = 0.5). The same significant difference appeared in the control and grafted mice groups (Figure 7B). Interestingly, no significant difference was detected in all studied groups whatever their origin (Figure 7C). In particular, no variation was seen in pups from grafted mice receiving immature or adult ovaries nor fresh or cryopreserved ovaries. In addition to the *H19* ICR, we evaluated the methylation status of the maternally methylated *Lit1* KvDMR1. A similar analysis was performed using *SmaI-ApaI* digested DNA (0.9 kb paternal and 2.6 kb maternal fragments) hybridized with a *Lit1* internal probe, on a more limited number of samples (Figure 7D). Again, a difference was observed between different tissues with a higher methylation index in the tongue compared to muscle and kidney. However, the global methylation status of the same three tissues showed no significant difference in all studied groups whatever their origin (Figure 7E).

## Discussion

In women, cancer therapy (chemo and radiotherapy) or myeloablative treatment required for stem cell transplantation before puberty may lead to a precocious ovarian failure. Today, the only option to avoid this detrimental side effect is ovarian cryopreservation of cortex slices from one ovary before treatment. Although the feasibility of surgery [48] and of techniques of ovary cryopreservation have been well documented, reimplantation of ovaries cryopreserved before puberty has not been performed in non pubertal recipients humans nor in animal models. As ovarian tissue banking in pediatric clinical practice is an ongoing process in some countries [13], [49], this represents potential benefit for patients at high risk of infertility. It is thus mandatory to define the optimal modalities of reimplantation for these immature ovarian cortexes. Results in adults in term of pregnancy remain promising, with four births of normal babies using cryopreserved ovaries [14], [15], [17], [18] and six pregnancies using fresh cortex [19]–[21]. The first report of pregnancy in a patient treated for Hodkgin's lymphoma had raised polemics on the authenticity of complete ovarian failure [50]–[52] and the possibility of spontaneous pregnancy in one such patient [53]. Another spontaneous pregnancy has been described in a patient treated for Hodgkin's disease after heterotopic reimplantation of cryopreserved ovarian cortex [54]. Our report is focused on the specific problem of pediatric patients as the few publications dealing with immature ovaries in mice only reported on the status of the primordial follicle pool [22], [55].

By using adult grafts, we confirm the results previously reported by others for fertility [56]. However, we demonstrate that the fertility of ovariectomized non-pubertal recipient can be restored by grafting immature ovaries capable of ovulation whatever the age of the recipient and the graft status (fresh or cryopreserved). These results may be explained by the high density of primordial follicles in ovarian cortex taken from young donors, as they are known to better withstand cryopreservation than more developed follicles [57]. Our results support the current clinical protocol which promotes ovarian cryopreservation in young girls undergoing sterilizing treatment before puberty.

Taking into account endocrine function, our model demonstrates that spontaneous puberty occurred when ovarian reimplantation was performed before puberty. A rescued cyclical hormonal activity was also observed when the recipients were young adults. Puberty induction has never been explored in animal models nor discussed in humans since hormonal substitution is available [58]. The risks of a long-term hormonal treatment initiated very early in life remain however undefined particularly in children who have an increased risk of secondary neoplasia. This higher risk is linked both to the potential genetic predisposition that may have given rise to the initial tumor and/or to the treatment received [59]. Using immature ovarian mouse grafts, even after cryopreservation and reimplantation before puberty (NNC group), we show that it is possible to induce puberty which in addition begins at a normal age. Reimplantation of immature cryopreserved cortex could thus be proposed to pre-pubertal girls to induce natural puberty but only if the tissue should be able to support long-term hormonal secretion and to assure fertility. Because all data indicate that the reproductive lifespan of grafted ovarian tissue in humans [60], [61] is limited, the main target of ovarian grafting is restoration of fertility and should therefore only be considered when relevant in this respect.

It is known that ovarian grafting, even without cryopreservation [62], induces a dramatic follicular loss (65%), whatever the age of donors and recipients (adult-adult or pre pubertal-adults [22]) or the location of graft (orthotopic or under kidney capsule [63]). This has also been reported in sheep [64] and in few cases of re-implanted cryopreserved human ovaries [65]. Our results confirm these data showing a 50% reduction of follicle density after transplantation independently of cryopreservation process and experimental group. Follicular loss may be related to the ischemia induced by the harvesting procedure and the prolonged delay of the neovascularisation following reimplantation [14]. Alternatively, a disruption of the granulosa-oocyte crosstalk, potentially induced by the procedure has been proposed [66]. The only models reporting no follicular loss used mouse fetal ovaries [23], [55], [67] that contain a high pool of primordial follicles more resistant to cryopreservation. The ischemia-related loss of the follicular population may account, in a large part, for the premature ovarian failure. Whereas immature ovaries contain a high pool of primordial follicles as compared to the adult ovaries, no gestation after 35 weeks of grafting was observed in our mouse model due to a complete depletion of follicles associated however to active steroidogenesis in interstitial cells. The interstitial cells are probably more protected from post grafting ischemia than the avascular granulosa cells, which rely on diffusion of oxygen and nutrients through the basal lamina. Although established in sheep models, to our best knowledge it is the first report that reveals the notion of premature failure of ovarian grafts in mice [64]. These data have to be considered to define the optimal period for reimplantation in young patients and exclude the use of the cryopreserved cortex for puberty initiation, favoring its consumption for pregnancy wishes.

Contrasting data have been reported on litter size, some authors describing normal [23], [68] whereas others suggest smaller litter sizes [69] similar to the results obtained in our study. Several reasons could be put forth to explain the decrease of pup number: decrease in the number of implantation sites related to reduced follicle recruitment per cycle or a misallocation of the ovarian graft or prenatal fetal loss. The unilateral graft could not be incriminated since there is no difference in litter size between unilateral ovariectomized mice and unmanipulated control groups. The number of implantation sites at day 5.5 of pregnancy as compared with the number of corpora lutea by ovary in the NNF group was significantly reduced suggesting a defect of embryo implantation. The risk of fetal loss because of malformations potentially related to epigenetic changes is a very important notion in view of treatment of human patients. Although epigenetic abnormalities in mice after assisted reproductive technologies have not been reported, (probably because such malformations lead to abortion), such abnormalities have been described after assisted reproductive technology in sheep [70]. Due to the increased frequency of developmental impairment such as BWS or Angelman syndrome [35], [36], [40], [71] or tumors such as retinoblastoma [31] in children born after ART (ovarian stimulation, *in vitro* fertilization and/or *in vitro* maturation of oocytes), this point should be discussed along with ovarian cryopreservation. The use of immature ovaries could increase this risk since the process occurs before the full establishment of imprinting in mature oocytes [34], [72].

Two DMRs from imprinted genes were analyzed, the paternally methylated *H19* DMR and the maternally methylated *Lit1* KvDMR1. Although the *H19* DMR is methylated in the male germline, this DMR was chosen because *H19* belongs to the cluster of genes affected in BWS. A second reason stems from the hypothesis that the maternally derived DMR must be protected from remethylation during the first stages of embryo development [73]. This protection could have been affected by the transplantation or cryopreservation of the immature ovaries. The *Lit1* KvDMR1 was analyzed because it clearly acquires its methylation through the female germline [74]. Interestingly, the methylation indexes in several tissues showed that the methylation profile of both DMRs is constant within an organ but varies significantly between tissues, i.e. muscle and tongue versus kidney for the *H19* DMR or muscle versus tongue for the KvDMR1. However, no significant difference was found between pups born from grafted ovarian or control females, independently of the graft status (fresh or cryopreserved) or of the age of recipients and donors. This strongly suggests that immature ovaries transplanted into immature or adult recipient females can produce oocytes which harbor a correct imprinting of at least the two *H19* and *LIT1* genes. These data should probably be taken with caution because of the relatively small number of samples. Furthermore, the reduced litter size observed in the cases of manipulated females could reflect spontaneous abortions due to malformations linked with imprinted genes. This is nevertheless an encouraging result.

In conclusion, our model supports the legitimacy to propose cryopreservation in young girls before gonadotoxic treatment as a tool to restore fertility, as has been done in adult women. Regarding our current knowledge concerning this procedure, one should remain cautious when delivering information to patients and their family at the time of cryopreservation, in terms of puberty induction and potential risks for children.
